# An Ultra-Stretchable Sensitive Hydrogel Sensor for Human Motion and Pulse Monitoring

**DOI:** 10.3390/mi12070789

**Published:** 2021-07-01

**Authors:** Bin Shen, Jiang Li, Yongtao Tang, Huihua Xu, Fengyu Li

**Affiliations:** Guangdong Provincial Key Laboratory of Functional Supramolecular Coordination Materials and Applications, Guangdong Engineering & Technology Research Centre of Graphene-Like Materials and Products, College of Chemistry and Materials Science, Jinan University, Guangzhou 510632, China; binshen@stu2018.jnu.edu.cn (B.S.); lijiang33@stu2020.jnu.edu.cn (J.L.); water@stu2019.jnu.edu.cn (Y.T.)

**Keywords:** flexible sensor, conductive hydrogel, sensitivity and linearity, motion and pulse monitoring

## Abstract

Ionic hydrogels with intrinsic conductivity and stretchability show great potential in flexible electronics. However, it remains a great challenge to achieve hydrogels with mechanical stretchability, ionic conductivity, optical transparency, and a self-healing ability at the same time. In this paper, we developed a hydroxyethylidene diphosphonic acid (HEDP) assisted poly(vinyl alcohol) (PVA) composite hydrogel to achieve high-performance stretch-sensitive sensor. Through a facile freeze–thaw strategy, the hydrogel could achieve large stretchability (up to 950% strain), good conductivity (10.88 S/m), excellent linear sensitivity (GF = 2.72, within 100% strain), high transparency, and significant self-healing ability. The PVA-HEDP hydrogel-based strain sensor is capable of monitoring various human movements from small scale (e.g., laryngeal vibration while speaking) to large scale (e.g., knee joint movement). Moreover, the multisite sensor array is capable of detecting the subtle differences between the pulse wave features from Cun, Guan and Chi positions, mimicking the three-finger palpation in Traditional Chinese Medicine. This work demonstrates that the composite hydrogel-based flexible sensor provides a promising solution for multifunctional human activities and health monitoring.

## 1. Introduction

With the blooming development of soft electronics, flexible strain sensors with highly stretchable, transparent, and self-healing characteristics have attracted extensive research interests due to applications such as human activity and health monitoring [1,2], electronic skin (e-skin) [3] and human–machine interaction [4,5]. Various strain sensor designs have been proposed to meet the specific requirements of the applications. For example, traditional conductive nanomaterials, such as metal nanowires, and low-dimensional carbon materials, have been engineered in an elastic insulating matrix (i.e., PDMS) to enable stretchable strain sensors [6,7,8]. However, the composite materials strategy imposes complicated procedures on the fabrication of the strain sensors with limited stretchability (usually less than 100%), transparency, and self-healing ability. New materials strategies are yet to be designed to address this issue.

Intrinsically conductive stretchable materials (e.g., ion-conductive hydrogel) provide a promising solution with a simple fabrication process, mechanical robustness, strain sensitivity, transparency, and a self-healing ability. Recently, ion conductive hydrogels have been intensively studied for the application in soft electronics such as flexible circuits [9], human motion detection [10], electronic skin [11], and biological tissue engineering [12]. For instance, in order to achieve high conductivity (146 mS/cm) at subzero temperatures, Liu et al. [13] introduced zwitterions into the polymer network to fabricate the electrolyte, while the fracture strength of the hydrogel is less than 0.005 MPa. And Chen et al. [14] synthesized a series of double-network and ion-conductive (10.3 S/m) self-healing hydrogels for circuit recovery, but they possessed low elasticity (0.02 MPa). Similarly, jiang et al. [15] committed to preparing a transparent conductive (3.2 S/m) organohydrogel for motion monitoring, but without the self-healing ability. In short, the electrical capabilities, mechanical properties, and optical performance seem to always ebb and flow. The key to the problem lies in the rational design of the conductive network and the construction of a synergistic response mechanism to balance various properties according to actual needs. 

In this paper, we explored the potential of introducing Hydroxyethylidene diphosphonic acid (HEDP) to Poly(vinyl alcohol) (PVA) to achieve tunable conductivity, self-healing ability, transparency in hydrogel-based strain sensor. HEDP is a well-known corrosion inhibitor in industry [16]. We chose HEDP because the hydroxyl group-rich molecular structure offers the ability to form strong hydrogen bonds with Poly(vinyl alcohol) (PVA), meanwhile the phosphonic acid groups in HEDP lend the resulting hydrogel proton conductivity, which make HEDP a potentially ideal conductive filler in hydrogel. The mechanical robustness and conductivity of the PVA-HEDP hydrogels can be tuned by controlling the concentration of HEDP. Through a convenient freeze–thaw strategy, we achieved hydrogels with excellent stretchability (up to 950% strain), great conductivity (10.88 S/m), good linear sensitivity (GF = 2.72, within 100% strain), high transparency, and great self-healing ability. Owing to these great properties, the hydrogel-based sensors showed outstanding performance in human activity and health monitoring, such as various human motion monitoring (which needs a wide-range stretchability) and epidermal pulse detection (which needs high sensitivity).

## 2. Materials and Methods

### 2.1. Materials

Poly(vinyl alcohol) (PVA, degree of hydrolysis: 98%) and hydroxyethylidene diphosphonic acid (HEDP, 60 wt% in water) solutions were purchased from Aladdin Chemical Reagent Co., Ltd. (Shanghai, China). Direct blue 86, sodium fluoresce and rhodamine B were provided by Maclin Chemical Reagent Co., Ltd. (Guangzhou, China). The deionized water was purified by a Milli-Q purifier. All the other reagents were purchased from Sigma-Aldrich, Ltd. and used without further purification.

### 2.2. Preparation of PVA-HEDP Hydrogel-Based Devices

PVA, HEDP were firstly dissolved in deionized water (the ratio of reagents is shown in Table 1). The mixture was stirred for 2 h at 105 °C to obtain a homogeneous solution. Then, the blended solution was cooled down to room temperature, and yielded the sol state of PVA-HEDP. After the sol was poured into different molds at −18 °C overnight, the PVA-HEDP hydrogel could be achieved. Hydrogel-based devices of different sizes can be obtained through various molds. The red, blue, and yellow PVA-HEDP-15 hydrogels were prepared by adding rhodamine B (1.5 mg·mL^−1^), direct blue 86 (1.5 mg·mL^−1^) and sodium fluorescein (1.5 mg·mL^−1^), respectively.

### 2.3. Characterization of PVA-HEDP Hydrogel

The Fourier transform infrared (FTIR) spectrum on 2 mm film samples were recorded on the *Frontier* instrument at 20 °C. PVA-HEDP homogeneous sol solutions were spin-coated onto a 1.5 mm-thick glass slide and frozen at −18 °C overnight. After thawing, the hydrogels were characterized by X-ray diffraction (XRD) measurement with a *Miniflex* 600 (Cu Kα radiation, 2θ = 5−60°, scan rate of 5° min^−1^). Ultraviolet-visible (UV-vis) spectra were recorded on an UV759CRT spectrophotometer (Shanghai Youke Instrument) in the wavelength range from 400 to 700 nm, with a resolution of 1 nm and a quartz cuvette as the substrate.

The tensile tests of hydrogel belts (length 60 mm, width 20 mm, and thickness 3 mm) were implemented on an AG-1 mechanical instrument with a speed of 50 mm·min^−1^ at room temperature. The compressive tests of hydrogel cylinders (diameter 30 mm and height 70 mm) were implemented on an AG-1 mechanical instrument with a speed of 10 mm min^−1^. The response of the sensor was tracked by the normalized change of resistance, ΔR/R_0_, where R_0_ and ΔR are defined as the original resistance and the resistance change upon stretching, respectively. The conductivity (σ) was recorded with the LCR meter IM3536 and the specific calculation method is as follows:

σ = L/(R·A)

where L, R and A are defined as the length, resistance and area of samples respectively.

## 3. Results and Discussion

**Figure 1:**
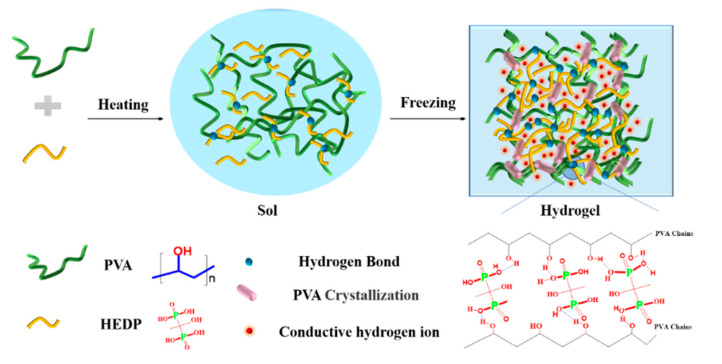
Scheme for the formation of the PVA-HEDP hydrogels by only one freeze–thaw cycle.

**Figure 2:**
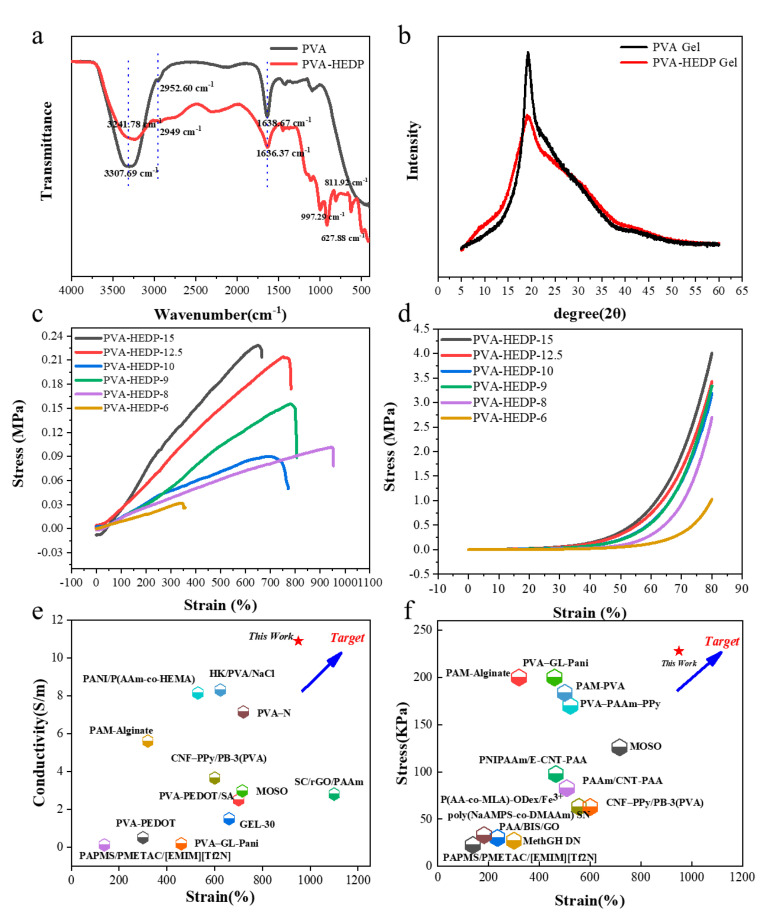
Characteristics of PVA-HEDP hydrogels. (**a**) FTIR spectra for the pure PVA gel and the PVA-HEDP gel. (**b**) XRD spectra for the pure PVA gel and the PVA-HEDP gel. (**c**) Stress–strain curves of the hydrogels with various HEDP contents. (**d**) Compressive stress–strain curves of the hydrogels with various HEDP contents. (**e**,**f**) Comparison of electrical and mechanical properties with previous research work [18,19,20,21,22,23,24,25,26,27,28,29,30,31,32,33].

We adopted a facile one-pot method to synthesize the water-based PVA-HEDP hydrogel. As shown in Figure 1, the homogeneous solutions comprising of PVA and HEDP with different mass ratios in water (labeled as PVA-HEDP-15, PVA-HEDP-12.5, PVA-HEDP-10, PVA-HEDP-9, PVA-HEDP-8 and PVA-HEDP-6) (Table 1) were placed at −18 °C overnight. After one rapid freeze–thaw cycle of the homogeneous sols, the PVA-HEDP hydrogels were produced. FTIR spectra of the pure PVA and PVA-HEDP gel (Figure 2a) showed the typical symmetrical stretching vibration of the hydroxyl group for the PVA gel at 3307 cm^−1^ and the PVA-HEDP gel at 3241 cm^−1^. And X-ray diffraction (XRD) characteristics clearly illustrate the formation of hydrogel crystallization peak (Figure 2b). Crystallization behavior of PVA chains would be weakened by increasing the HEDP content of the mixture, due to the strong gelatinization of HEDP molecules enlarging the space between PVA chains [17]. Therefore, compared with pure PVA gel, PVA-HEDP has a lower crystallization peak at about 20°. FTIR and XRD show that the gelation mechanism of PVA-HEDP hydrogel is mainly deduced by hydrogen bonding and PVA crystallization. HEDP promotes the gelation of the hydrogel, and the crystallization of PVA chains enhances the mechanical strength of the hydrogel.

To be used as an active material in a strain sensor, the hydrogel should sustain a large degree of stretching and accordingly yield continuously varied conductivity, thus maintaining good sensing sensitivity in a long strain range. By changing the concentration of HEDP, it was found that the mechanical properties of the hydrogel can be tuned. The tensile modulus and compression modulus increased to 0.228 MPa and 4 MPa as the concentration of HEDP increased to 21.4 wt% (Figure 2c,d). A maximum stretchability (up to 950%) was achieved with a moderate HEDP concentration. The tunable mechanical properties were attributed to the noncovalent interactions such as dynamically dissociated and reassociated hydrogen bonds formed between HEDP and PVA molecules, which disrupt the interaction between the PVA chains, and function as sacrificial bonds to effectively dissipate energy before the breaking of PVA chains during mechanical deformation. With the HEDP concentration increases, the hydrogel of PVA-HEDP-6, PVA-HEDP-8, PVA-HEDP-9, PVA-HEDP-10, PVA-HEDP-12.5 and PVA-HEDP-15 show conductivity of 9.33 S/m, 10.88 S/m, 9.36 S/m, 9.32 S/m, 9.68 S/m, and 9.88 S/m, respectively. All the PVA-HEDP hydrogels with different loading ratios of HEDP exhibited impressive conductivity. However, it is worth noting that the conductivity is not linearly related to the ratio of HEDP. This is because the conductivity of the hydrogel is determined by the H^+^ migration in the polymer network. While higher HEDP ratio is capable of providing more free H^+^, it tends to produce a denser cross-linked hydrogel network, which will in turn hinder the free migration of H^+^. Thus, we need to find a balance between conductivity and mechanical properties by tuning the ratio of HEDP according to specific application requirements. We further compared our results with previous studies that demonstrate hydrogels with excellent mechanical toughness or conductivity [18,19,20,21,22,23,24,25,26,27,28,29,30,31,32,33], as shown in Figure 2e,f. It is also worth mentioning that our PVA-HEDP hydrogels exhibited excellent conductivity and mechanical performance simultaneously. 

**Figure 3:**
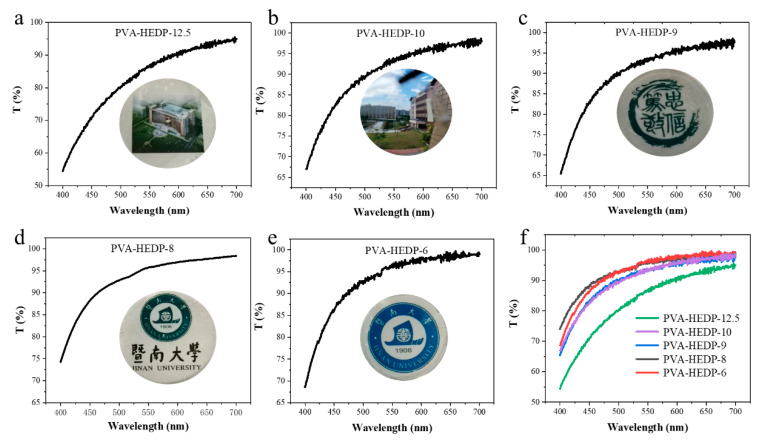
UV−vis spectra and graphs of PVA-HEDP hydrogels with different HEDP content. (**a**) PVA-HEDP-12.5, (**b**) PVA-HEDP-10, (**c**) PVA-HEDP-9, (**d**) PVA-HEDP-8, (**e**) PVA-HEDP-6, (**f**) transparency of the hydrogels with different HEDP content.

In addition to the excellent mechanical deformability and ion conductivity, our PVA-HEDP hydrogels show high optical transparency after the freeze–thaw cycle, suggesting the unfrozen state of the hydrogels. As shown in Figure 3, the transmittance of PVA-HEDP hydrogel is higher than 50% at wavelengths of 400–700 nm. And the transmittance of PVA-HEDP-10, PVA-HEDP-9, PVA-HEDP-8 and PVA-HEDP-6 even exceed 90% at wavelengths of 500–700 nm. Moreover, due to the high transparency, we can clearly see complex patterns, such as the campus scenery, characters, numbers and text through the hydrogel films. It was also found that the transparency increased with the HEDP content. We know that the transparency of the PAV-based hydrogels is determined by the size and distribution of the crystalline areas formed at subzero temperature. The enhanced transparency of PVA-HEDP hydrogels is attributed to the key role of HEDP in forming strong hydrogen bonds with PVA molecules and meanwhile impeding the hydrogen bonding between PVA chains, thus reducing the volume of the crystalline region in the hydrogel network. 

**Figure 4:**
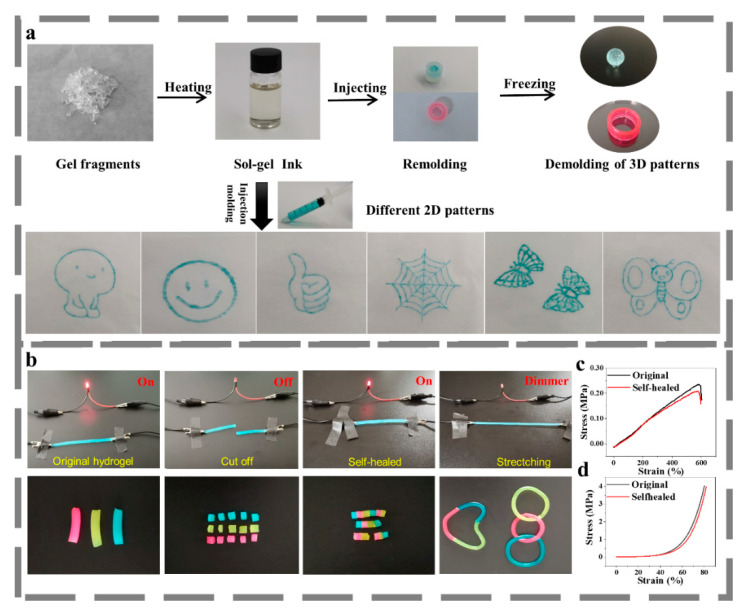
Thermoplastic performance and self-healing ability of PVA-HEDP hydrogels. (**a**) Thermoplastic behavior of PVA-HEDP hydrogels, (**b**) Self-healing process of the hydrogel, (**c**) Tensile stress–strain curves of PVA-HEDP hydrogel before and after self-healing, (**d**) Compressive stress-strain curves before and after self-healing.

Self-healing ability is another highly anticipated property for hydrogel to work in flexible strain sensors requiring dynamic shape changing and high strain values, which can lead to potential deformation or even structural failure. We explored the self-healing behavior of the PVA-HDEP hydrogel by a simple heating–freezing process. We conducted the heating–shaping experiment by cutting the hydrogel into fragments, and putting them into a glass vial. After heating at 90 °C for about 30 min, the hydrogel fragments transformed into a transparent and homogenous sol state and were readily to form new shapes by remolding. They were then frozen at −18 °C overnight. As shown in Figure 4a, the sol can be poured into desired three-dimensional molds or extruded through a syringe to draw new patterns. Then, the conductance and self-healing properties of the PVA-HDEP hydrogel were investigated by connecting the gel in a circuit in series with a LED indicator (Figure 4b). In the original state of the PVA-HEDP hydrogel, it could act as a conductor to light the LED. After cutting the hydrogel into two parts, the LED indicator was powered off. When the two hydrogel parts were reconnected at the broken surface and treated with the aforementioned heating–freezing process, the hydrogel was self-healed completely and could be employed as conductor to light the LED again. Furthermore, the self-healed hydrogel was capable of withstanding large-scale stretching, without losing the conductance. The repaired blue, yellow and red hydrogels (stained by direct blue 86, fluorescein sodium and rhodamine B, respectively) can also complete the reintegration of color and device morphology. These phenomena, as well as the tensile and compressive strength of the hydrogels before and after healing (Figure 4c,d), indicate the self-healing of both the electrical and mechanical characteristics. The fundamental self-healing mechanism is the dynamic dissociation caused by heating and the re-association of crystalline regions and hydrogen bonds determined by freezing [34].

**Figure 5:**
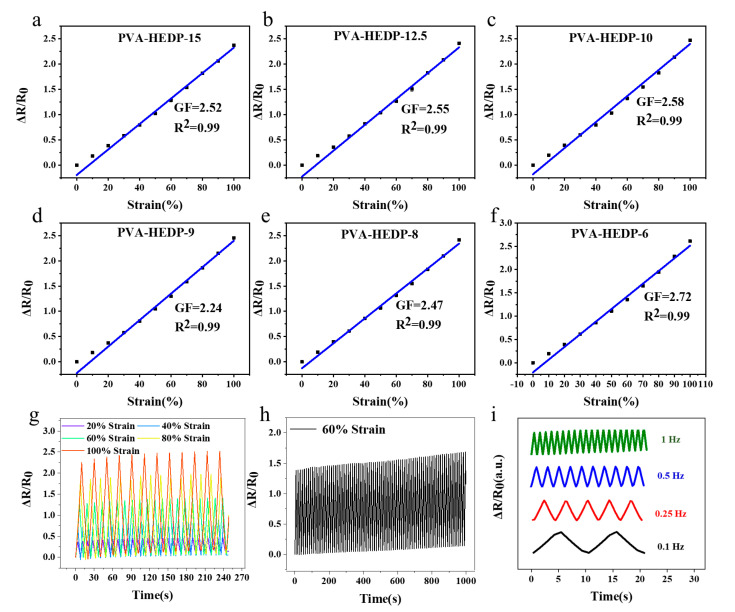
Resistance–strain curves of PVA-HEDP hydrogels with different HEDP content. (**a**) PVA-HEDP-15, (**b**) PVA-HEDP-12.5, (**c**) PVA-HEDP-10, (**d**) PVA-HEDP-9, (**e**) PVA-HEDP-8, (**f**) PVA-HEDP-6. (**g**) Response of the hydrogel to cyclic loading–unloading of 20%–100% strain. (**h**) Response of the hydrogel to cyclic loading–unloading of 60% strain for 1000 s, showing the stability of the strain sensor. (**i**) Frequency response curves of the PVA-HEDP hydrogel-based sensor at applied strain frequencies from 0.1 to 1 Hz under 10% strain.

To be used as active material in a strain sensor for E-skin application, the hydrogels should possess high sensitivity in a wide linear range and maintain a stable and repeatable resistance reading under stretching. To test these properties, we fabricated strain sensors based on the PVA-HEDP hydrogels, and evaluated the electromechanical properties of the sensors by monitoring their relative resistance change, ∆R/R_0_, during stretching, where ∆R is the resistance variation in response to strain loading and R_0_ is the original resistance of the sensor without strain loading. Figure 5a–f shows the relative resistance change–strain curves, where the slopes of the curves represent the sensor sensitivity. It is found that the PVA-HEDP hydrogels all exhibit a linear region in the strain range of 0–100%, with the gauge factor (GF) from 2.24 to 2.72, which is an indicator of high sensitivity. This GF exhibited by our PVA-HEDP hydrogels is higher than that of previously reported hydrogels [15,35]. Note that the PVA-HEDP hydrogels shown in Figure 5a–f were fabricated from the same 3D mold, while varying the mass ratios of PVA, HEDP and water. The results indicate that the conductive properties of the composite hydrogel can be tuned by controlling the concentrations of PVA and HEDA. For further comparison, we also fabricated two PVA-HEDP-15 sensors from identical molds, and compared their responsivity to cyclic strain loading–unloading over time (Figure S1). It can be seen that two sensors show almost the same output signals, demonstrating good reproducibility of the hydrogels. In addition to high sensitivity and linearity, the sensor also showed good repeatability in the detection of moderate and high strains from 20% to 100% (Figure 5g). The cycle test of the sensor shown in Figure 5h further demonstrates reproducible response under continuous cycles of 60% stretching–unstretching. Furthermore, as shown in Figure S2, the sensor generated a stable output signal during the loading–unloading of 100% strain over 700 s. The reproducible and stable response demonstrate the high repeatability, stability and durability of the sensor. Finally, we also measured the electrical response of the PVA-HEDP hydrogel-based sensor at various applied frequencies from 0.1 Hz to 1 Hz under 10% strain, and the results are shown in Figure 5i. The responsive ∆R/R_0_ signals follow closely with the input strain, and no apparent delay is observed within the frequency range studied, indicating that the hydrogel is sensitive and stable in the frequency range from 0.1 Hz to 1 Hz, suitable for human motion and pulse monitoring. The high sensitivity and linearity, coupled with the wide detection range of strain enable the PVA-HEDP hydrogel-based strain sensor to detect a wide range of human activities. This, together with the additional advantages of mechanical robustness, high transparency, and the self-healing ability of the hydrogel mentioned above, make the PVA-HEDP hydrogel-based strain sensor a competitive one for human motion and health monitoring. 

**Figure 6:**
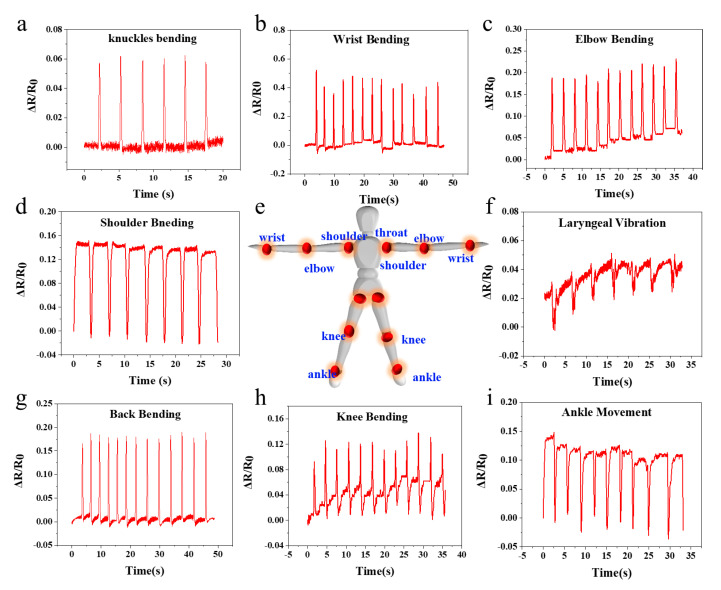
PVA-HEDP hydrogel sensor for human joint motion monitoring. (**a**) Knuckles bending, (**b**) wrist bending, (**c**) elbow bending, (**d**) shoulder bending, (**e**) schematic diagram of the movable joints in the human body, (**f**) laryngeal vibration, (**g**) back bending, (**h**) knee movement, (**i**) ankle movement.

With excellent mechanical toughness and sensitivity of the PVA-HEDP hydrogel-based sensors, we may explore the application of the sensors in monitoring various human motions (Figure 6). As a bridge between bones, moving joints (Figure 6e) are closely related to human motion. We first attached the sensors to a finger to investigate the electrical response of the sensor to repeated knuckle bending with a frequency of ~0.4 Hz. As shown in Figure 6a, with the finger bending, the resistance increased rapidly and sharply, which is attribute to the stretching of the hydrogel. With the relaxing of the hydrogel upon the relaxation of the finger, the resistance signal recovered rapidly and completely, accordingly. Moreover, the sensor exhibited stable and repeatable electrical response in the cycling test with a low noise level. Due to the high sensitivity, the sensor is capable of detecting tiny signals produced by small-scale movements, e.g., laryngeal vibration while speaking “Jinan University”, shown in Figure 6b. In addition, the sensor is also able to monitor larger-scale human motions, such as repeated bending of shoulder, back, elbow, ankle, wrist and knee, all with stable and reproducible response signals. The larger-scale motion produces higher signal levels than the smaller-scale motion, and the signal shows different characteristics according to the type of joint movement, indicating the capability of the sensor to distinguish between human motions of different scales and movements.

**Figure 7:**
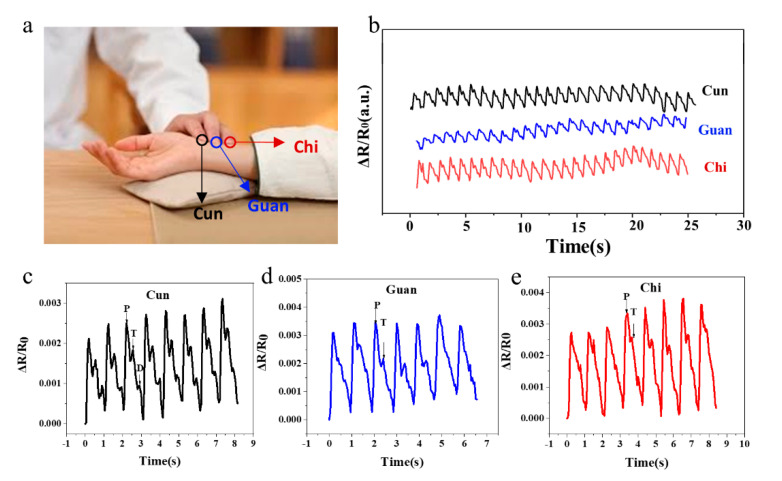
PVA-HEDP hydrogel sensor for pulse detection. (**a**) A photo of TCM pulse diagnosis, (**b**) Cun, Guan and Chi pulse waveforms of a 25-year-old male subject, (**c**–**e**) Measured typical pulse waveforms at the (**c**) Cun, (**d**) Guan, (**e**) Chi positions respectively.

Inspired by pulse diagnosis in traditional chinese medicine (TCM), that is, TCM doctors diagnose patients by detecting the pulse at corresponding points on the radial artery defined as Cun, Guan and Chi positions [36] (Figure 7a), we designed a three-channel PVA-HEDP hydrogel-based sensor array mimicking the three fingers of a TCM doctor to record the pulse waves. The typical Cun, Guan and Chi pulse waveforms of a 25-year-old male subject are shown in Figure 7b. Generally, the shapes of the pulse waves from three positions are similar, indicating a heart rate of 65 beats-per-minute. However, the Cun position shows three typical peaks, namely percussion wave (P), tidal wave (T) and dicrotic wave (D) (Figure 7c), and the Guan and Chi positions show obvious P wave while T and D wave signals are relatively weak (Figure 7d,e). Due to the high sensitivity, such hydrogel-based sensors can differentiate the key characteristics of the Cun, Guan and Chi pulse, which is believed to relate to the health condition of human organs. 

## 4. Conclusions

In summary, we explored the potential of introducing HEDP to PVA to realize composite PVA-HEDP hydrogels for high performance strain sensors. We found that the composite material strategy allows the formation of strong hydrogen bonds between HEDP and PVA, thus ensuring ultra-stretchability and tunable conductivity of the hydrogels. More importantly, it allows us to tune the overall properties of the hydrogel by tuning the composition. Through composition optimization, we achieved hydrogels with large stretchability (up to 950% strain), good conductivity (10.88 S/m), excellent linear sensitivity (GF = 2.72, within 100% strain), high transparency, and significant self-healing ability. With these properties, we realized the hydrogel-based sensors and a multisite sensor array for monitoring of various human motions from small scale (e.g., laryngeal vibration while speaking) to large scale (e.g., knee joint movement) and pulse waves at Cun, Guan and Chi positions. Given the above attributes, the PVA-HEDP hydrogel-based strain sensor can be easily implemented in various wearable devices, providing a promising solution for low-cost human activities and health related monitoring.

## Figures and Tables

**Table 1:** Different proportions of PVA-HEDP hydrogels.

Hydrogel	PVA(g)	HEDP (mL)	H_2_O (mL)
PVA-HEDP-15	3	10	15
PVA-HEDP-12.5	3	12.5	12.5
PVA-HEDP-10	3	15	10
PVA-HEDP-9	2.5	12	9
PVA-HEDP-8	2	12	8
PVA-HEDP-6	2	12	6

## Supplementary Materials

The following are available online at https://www.mdpi.com/article/10.3390/mi12070789/s1, Figure S1: Response of the PVA-HEDP-15 hydrogel sensors fabricated from two identical molds to cy-clic loading-unloading of 20% strain. Figure S2: Response of the hydrogel to cyclic load-ing-unloading of 100% strain.
