# Supplementary material for: An Ultra-Stretchable Sensitive Hydrogel Sensor for Human Motion and Pulse Monitoring
Source: Micromachines (Basel). 2021 Jul 1;12(7):789. doi: 10.3390/mi12070789 (PMC8305011; doi:10.3390/mi12070789)
Supplement: Supplementary file 1 [file micromachines-12-00789-s001.zip › micromachines-1275293-supplementary.pdf]

Supporting Information

An Ultra-stretchable Sensitive Hydrogel Sensor for Human Motion and Pulse Monitoring

Bin Shen, Jiang Li, Yongtao Tang, Huihua Xu* and Fengyu Li*

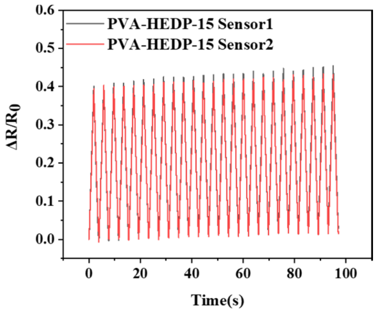

Figure S1. Response of the PVA-HEDP-15 hydrogel sensors fabricated from two identical molds to cyclic loading-unloading of 20% strain.

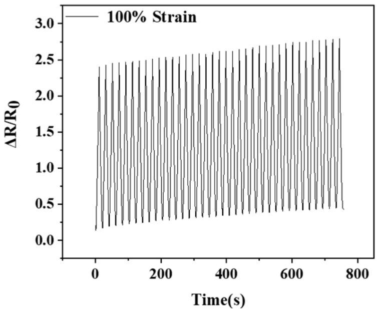

Figure S2. Response of the hydrogel to cyclic loading-unloading of 100% strain.
